# The chicken cornea as a model of wound healing and neuronal re-innervation

**Published:** 2011-09-21

**Authors:** Eric R. Ritchey, Kimberly Code, Christopher P. Zelinka, Melissa A. Scott, Andy J. Fischer

**Affiliations:** 1 College of Optometry, The Ohio State University, Columbus, OH; 2 School of Allied Medical Professions, The Ohio State University, Columbus, OH; 3 Department of Neuroscience, The Ohio State University, Columbus, OH

## Abstract

**Purpose:**

The cornea is the major refractive component of the eye and serves as a barrier to the external environment. Understanding how the cornea responds to injury is important to developing therapies to treat vision disorders that affect the integrity and refractive properties of the cornea. Thus, investigation of the wound healing responses of the cornea to injury in a cost-effective animal model is a valuable tool for research. This study characterizes the wound healing responses in the corneas of White Leghorn chicken.

**Methods:**

Linear corneal wounds were induced in post-natal day 7 (P7) chicks and cellular proliferation, apoptosis and regulation of structural proteins were assessed using immunohistochemical techniques. We describe the time course of increased expression of different scar-related markers, including vimentin, vinculin, perlecan and smooth muscle actin.

**Results:**

We find evidence for acute necrotic cell death in the corneal region immediately surrounding cite of incision, whereas we failed to find evidence of delayed cell death or apoptosis. We find that the neuronal re-innervation of SV2-positive axon terminals within the corneal stroma and epithelium occurs very quickly after the initial scarring insult. We describe an accumulation of cells within the stroma immediately underlying the scar, which results, at least in part, from the local proliferation of keratocytes. Further, we provide evidence for scar-induced accumulations of CD45-positive monocytes in injured corneas.

**Conclusions:**

We conclude that the chick cornea is an excellent model system in which to study wound healing, formation of scar tissue, and neuronal re-innervation of sensory endings.

## Introduction

The cornea is a stratified, transparent, avascular tissue which acts as a barrier to the external environment and serves as the major refractive component of the eye. The cornea is derived from 2 sources: the ectoderm, which provides the corneal epithelium and stroma, and neural crest cells, which provide keratocytes and endothelial cells [1] (reviewed by [2]). In the mature cornea, corneal keratocytes remain quiescent until the introduction of an insult, which leads to a cascade of cell-cell signaling and wound healing response (reviewed by [3,4]). A response to insult that involves production of scar tissue into the cornea can compromise the optical properties of the cornea. Thus, the ability of the cornea to heal from damage resulting from infection or trauma without introducing excessive scarring is vital to maintaining visual function.

There are multiple different animal models that are used to examine corneal wound healing in response trauma or surgery [5-12]. One animal model that has received relatively little attention in corneal wound healing is the chicken. The chicken cornea has several advantages compared to rodent models of wound healing: 1) chickens have much larger eyes and corneas (approximately 9 mm in diameter and 400 µm in thickness) than rodents, making these eyes more amenable to experimental manipulations such as refractive surgical procedures, 2) the intraocular lens in the chicken is much smaller than in the rodent model, allowing for delivery of intravitreal injection of compounds such as BrdU without complication 3) newly hatched chicks are inexpensive, 4) unlike rodents, chicken corneas have a true Bowman’s membrane and the corneal layers are proportional to the human cornea. The chick cornea is composed of 5 layers similar to the human cornea, and the cellular composition and the proportional thickness of the different layers are comparable to those of the human cornea [13]. Given the similarities between chicken and human corneas, the chicken model represents a useful animal model for examining wound healing.

Currently, the scientific literature focuses on the response of the chick cornea to refractive surgery techniques such as photorefractive keratectomy (PRK) and laser in situ keratomileusis (LASIK) [14-16]. The purpose of this study was to characterize the wound healing process in chick cornea following induced trauma. We assess the time-course of cell death, cellular proliferation, and neuronal re-innervation. In addition, we characterize the patterns of expression of structural proteins that are known to be associated with corneal healing.

## Methods

### Animals

Animals were used in accordance with international standards for animal treatment established by the National Institutes of Health, ARVO and The Ohio State University. Newly hatched white leghorn chickens (*Gallus gallus domesticus*) were obtained from the Ohio State University Department of Animal Sciences (Columbus, OH) and raised on a cycle of 12 h light, 12 h dark (light 7 AM to 7 PM) in a stainless steel brooder. Chicks were fed Purina chick starter (Purina, St Louis, MO) and water ad libitum.

### Intraocular injections and corneal wound generation

Animals were anesthetized by inhalation of 2.5% isoflurane in O_2_ at a flow rate of 1.5 l/min. Corneal anesthesia was achieved using 1 drop of 0.5% topical proparacaine ophthalmic solution. A 4 mm Barraquer pediatric lid speculum was inserted and the chick placed under a Leica S6E stereo microscope (Leica, Buffalo, NY). A single linear corneal incision was made, nasal to temporal, using a Nichamin LRI (Storz, Rochester NY) diamond knife set for a blade depth of 0.50 mm through the central cornea with a mid-stroma target depth. After incision, 1 drop of 0.3% moxifloxacin topical ophthalmic solution (Vigamox; Alcon, Fort Worth, TX) was applied and animals were subsequently monitored for signs of corneal infection.

Cellular proliferation was assayed by using intravitreal injections of the thymidine analog 5-bromo-2-deoxyuridine (BrdU). Six μg of BrdU diluted in 20 μl sterile saline was injected into the dorsal quadrant of each eye through the superior eyelid.

### Tissue dissection, fixation, and sectioning

Animals were sacrificed; the eyes were enucleated and hemisected along the equator of the eye. Anterior segments were fixed in 4% paraformaldehyde (PFA) + 3% sucrose in 0.1 M PB, pH 7.4 for 25 min at room temperature. Samples were washed twice for 15 min in 0.05M phosphate buffered saline (PBS) and cryoprotected in 20% sucrose in PBS overnight. Anterior segments were placed in Optimal Cutting Temperature (OCT) compound (Sakura Tissue Tek, Torrance, CA) medium and freeze-mounted onto sectioning blocks. Corneal sections, nominally 12 microns thick, were made transversely through the corneal wound using a Microm HR550 cryostat (Thermo Fisher, Walldorf, Germany). Sections were thaw-mounted on SuperFrost Plus slides (Thermo Fisher, Fairlawn, NJ) and stored at –20 °C until immunolabeling.

Corneal whole-mounts were obtained after the anterior segment was fixed in 4% PFA as described above. Corneas were dissected away from anterior segments. Isolated corneas were equilibrated in 20% sucrose in PBS and taken through 3 cycles of freezing and thawing. Cornea whole-mounts were permeabilized using graded washes of DMSO (40%→ 60%→ 80%→ 100%→ 80%→ 60%→ 40%). Individual corneas were placed in 400 μl of primary antibody solution (antiserum + PBS + 0.2% Triton X-100 + 0.01% NaN_3_) and placed on a nutating shaker overnight. The primary antibody solution was aspirated and corneas were washed two times in PBS. Corneas were covered with 400 μl of secondary antibody solution (Alexa Fluor [Invitrogen, Carlsbad, CA] antiserum + PBS + 0.2% Triton X-100 + 0.01% NaN_3_) and placed on a nutating shaker overnight. Slides were mounted under cover glass on 80% glycerol in water.

### Immunolabeling and photography

Slides were warmed to room temperature and ringed with rubber cement. Sections were washed two times in 0.05 M PBS for 10 min and covered with 250 μl of primary antibody solution (antiserum + PBS + 0.2% Triton X-100 + 0.01% NaN_3_ + 5% normal goat serum or normal donkey serum) and incubated for 24 h in a covered, humidified chamber at room temperature. The primary antibodies included: (1) mouse anti-BrdU used at 1:80 dilution (G3G4; Developmental Studies Hybridoma Bank [DSHB]; University of Iowa, Iowa City, IA), (2) mouse anti-CD45 used at 1:200 dilution (HIS-C7; Cedi Diagnostics, Prionics AG, Zurich, Switzerland), (3) mouse anti-α-smooth muscle actin (αSMA) used at 1:500 dilution (1A4; Sigma-Aldrich, St Louis, MO), (4) rat anti-substance P used at 1:400 dilution (Ab6338; Abcam, Cambridge, MA), (5) mouse anti-synaptic vesicles used at 1:1,000 dilution (SV2; DSHB), (6) mouse anti-vimentin used at 1:80 dilution (H5; DSHB), (7) mouse anti-vinculin used at 1:50 dilution dilution (VN3–24; DSHB), (8) mouse anti-perlecan used at 1:50 dilution (5C9; DSHB), and (9) rabbit anti-cleaved caspase 3 (CC3) used at 1:1,200 dilution (AF835; R&D Systems, Minneapolis, MN). The primary antibody solution was aspirated and slides were washed in two times in 0.05 M PBS for 10 min. Slides were covered with 250 μl of secondary antibody solution (antiserum + PBS + 0.2% Triton X-100 + 0.01% NaN_3_) in a covered, humidified chamber at room temperature for 1 h. Slides were washed in PBS and cover glass mounted on 4:1 glycerol to water (volume:volume). Antigen retrieval was used to permit immunolabeling for BrdU; sections were washed for 7 min in 4 M HCl. Secondary antibodies included donkey-anti-goat-Alexa488, goat-anti-rabbit-Alexa488, goat-anti-mouse-Alexa488/568, rabbit-anti-goat Alexa488 and goat-anti-mouse-IgM-Alexa568 (Invitrogen) diluted to 1:1,000 in PBS plus 0.2% Triton X-100.

We evaluated the specificity of primary antibodies by comparison with published examples of results and assays for specificity. None of the observed labeling was due to non-specific labeling of secondary antibodies or autofluorescence because sections labeled with secondary antibodies alone were devoid of fluorescence. Secondary antibodies included goat-anti-rabbit-Alexa488/568 and goat-anti-mouse-Alexa488/568 (Invitrogen) diluted to 1:1000 in PBS plus 0.2% Triton X-100.

### Labeling of dying cells

Dying cells were identified using Terminal deoxynucleotidyl Transferase Biotin-dUTP Nick End (TUNEL) Labeling (In situ Cell Death Kit TMR; Roche, Indianapolis, IN) according to the protocols provided by the manufacturer. To label cells with compromised plasma membranes we applied a single topical drop (50 µl; 0.2 mM in saline) of Ethidium Homodimer-1 (EtHD; Live/Dead® Cell Viability Assay; Invitrogen) shortly (<15 min) after incisions were made into the cornea. Fifteen min after the application of the EtHD the corneas were harvested and fixed, as described above.

### Microscopy, cell counts and statistics

Photos were taken using a Leica DM 5000B microscope and Leica DC500 12-megapixel cooled CCD camera. Confocal microscopy was performed using a Zeiss LSM 510 system (Zeiss, Thornwood, NY) at the Hunt-Curtis Imaging Facility at The Ohio State University. Images were optimized for color, brightness and contrast, and double-labeled images overlaid by using Adobe Photoshop^™^6.0 (Adobe, San Jose, CA). Cells counts were performed and statistical analysis completed using SPSS V17.0 (IBM, Inc, Armonk, NY).

## Results

### Cellular proliferation after corneal insult

**Figure 1:**
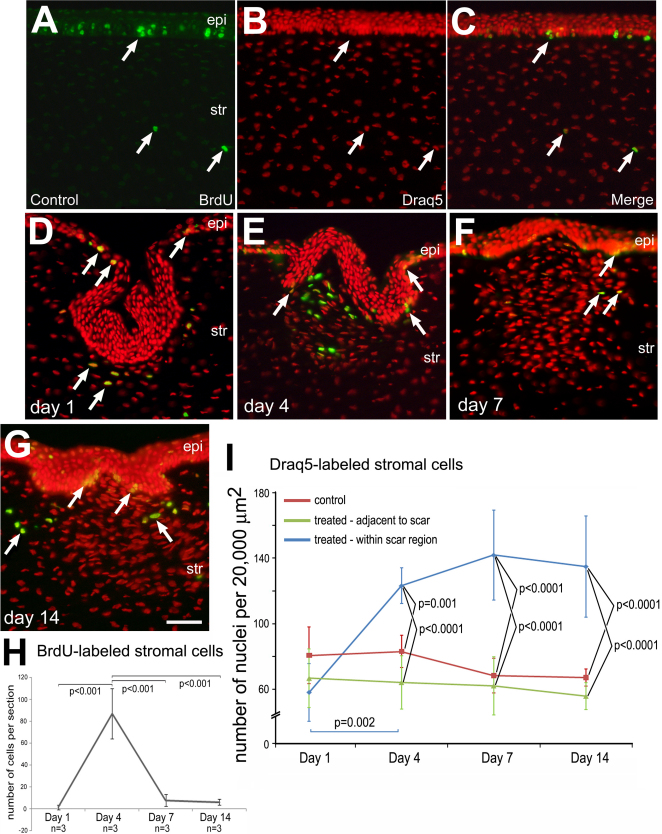
Corneal injury stimulates the proliferation of stromal cells. Transverse sections of the cornea were labeled for BrdU (green) and Draq5 (red nuclei). Uninjured corneas (**A**-**C**) and injured corneas were harvested (and BrdU injected) at day 1 (BrdU injected 4 h prior; **D**), day 4 (BrdU injected at days 1, 2, and 3; **E**), day 7 (BrdU injected at days 4 and 5; **F**), and day 14 (BrdU injected at days 7 and 8; **G**). Arrows indicate BrdU-labeled nuclei. **H**: Plot indicating the mean (±SD; n=3 for each time point) number of BrdU-labeled cells within 20,000 µm^2^ within the stroma within the region of the incision. The calibration bar (50 µm) in **G** applies to **A**-**G**. **I**: Plots indicating the mean (±SD; n≥5 for each time point) indicate the mean number of stromal nuclei per 20,000 µm^2^ in control, undamaged corneas (red), treated stromal region adjacent to the incision site (green), and treated stromal region within the incision site (blue). ANOVA was performed to determine significance of difference within the data set and a Bonferroni post-hoc comparison was performed to determine significance between and within experimental groups. There was no significant difference (p>0.05) over time among treatment groups, with the exception of between 1 and 4 days after injury within the site of incision (p=0.002; **I**). Abbreviations: epi – epithelium, str – stroma, endo – endothelium.

To identify newly generated cells in the cornea we injected BrdU into the vitreous chamber and harvested corneas at different times after BrdU-treatment. Corneas were harvested (and BrdU injected) at day 1 (BrdU injected 4 h prior), day 4 (BrdU injected at days 1, 2, and 3), day 7 (BrdU injected at days 4 and 5), and day 14 (BrdU injected at days 7 and 8). Under control conditions with no incision, BrdU-positive cells were observed near the basement membrane of the corneal epithelium (Figure 1A-C), consistent with the notion that mitotically active cells are normally found in basal epithelial layers. In addition, we occasionally observed BrdU-positive cells in the corneal stroma, consistent with the quiescent nature of keratocytes in the normal cornea (Figure 1A-C). After damage, proliferating and newly generated cells were observed in the stroma and epithelium immediately surrounding the corneal incision. One day after corneal incision, the wound was filled by a layer of epithelial cells that were negative for BrdU (Figure 1D). By comparison, BrdU-positive cells were observed at the ridge of the corneal epithelium leading into the trough caused by the incision (Figure 1D). Few BrdU-positive cells were observed in the corneal stroma immediately below the epithelium (Figure 1D). At 4 days after injury, there were no BrdU-positive cells in the epithelium overlying the corneal incision (Figure 1E). By comparison, there was a significant increase in BrdU-positive cells observed within an area of hypercellularity in the anterior stroma immediately underlying the site of incision (Figure 1E,H). At 7 days after injury, the number of BrdU-positive cells within the zone of injury decreased compared to that seen at 4 days after injury (Figure 1F,H). At 14 days after injury, many BrdU-positive cells were observed in basal layers of the epithelium in the region of the corneal incision (Figure 1G). In addition, at day 14, we observed a few BrdU-positive cells in the stroma at the edges of the incision site (Figure 1G,H).

Counts of Draq5-labeled nuclei in the corneal stroma were performed to assess the accumulation of cells following injury. In control corneas, the average density was 74.70 nuclei per 20,000 µm^2^ (n=20, SD±12.97). There were significant increases in cell density in the stroma after injury (Figure 1A-G,I). At 1 day after injury, the cell density within the wound-region of the stroma was not significantly different from that of control corneas (Figure 1D,I). At 4 days after injury, there was a significant, approximate twofold increase in cellular density in the stroma within the zone of injury (Figure 1E-I). The increased cell density that was seen at 4 days after injury was not significantly different at 7 and 14 days after injury (Figure 1F-G,I).

It is possible that the increased cell density of stromal cells in the corneal wound occurred due to cellular migration. Thus, we examined whether there was a depletion of cell density in the stromal regions immediately adjacent to the corneal injury. There was no significant difference in stromal cell density in uninjured corneas compared to the region adjacent to the injury for any time point (Figure 1I). Moreover, there was no significant change in cell density in the stromal region adjacent to the scar, at any time point after injury (Figure 1I).

### Expression of filamentous proteins after corneal insult

**Figure 2:**
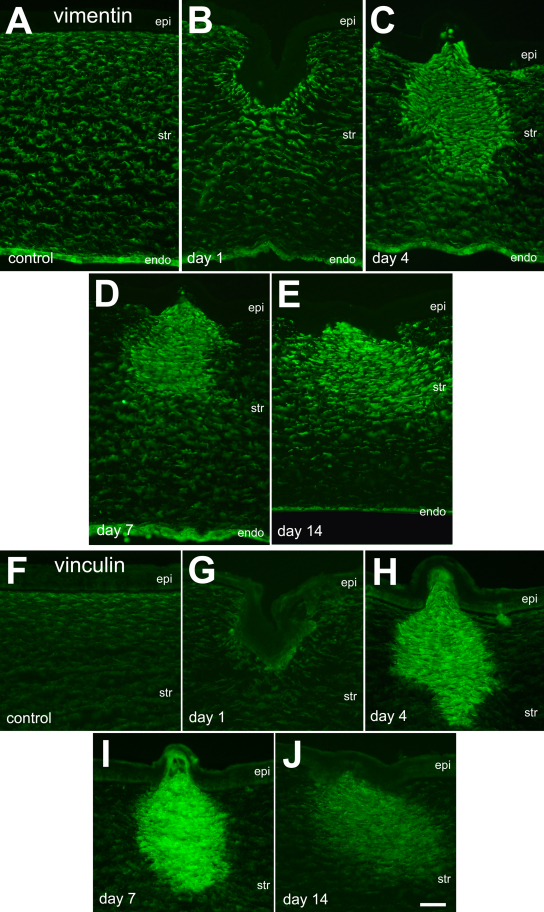
The filamentous proteins vimentin and vinculin are upregulated in stromal cells following corneal injury. Transverse sections through the cornea were labeled with antibodies to vimentin (**A**-**E**) or vinculin (**F**-**J**). Corneas were harvested from control eyes (**A** and **F**) or 1 day (**B** and **G**), 4 days (**C** and **H**), 7 days (**D** and **I**) and 14 days (**E** and **J**) after injury. The calibration bar (50 µm) in **J** applies to all panels. Abbreviations: epi – epithelium, str – stroma, endo – endothelium.

Following corneal incision, we found an upregulation of the intermediate filament vimentin in stromal cells. Control corneas had vimentin-immunoreactivity in most, if not all, stromal keratocytes (Figure 2A), similar to previous descriptions [17,18]. Vimentin was not observed in the corneal epithelial cells, whereas vimentin was observed in the endothelial cells (Figure 2A). At 1 day after corneal incision, vimentin appeared modestly upregulated in stromal cells directly under the corneal epithelium where Bowman’s membrane was disrupted (Figure 2B). At 4 days after corneal incision, vimentin-immunofluoresence was dramatically increased in stromal cells within the wound, and these cells extended several hundred micrometers into deeper layers of the corneal stroma (Figure 2C). In the stromal areas lateral to the site of incision, Bowman’s membrane appeared to remain intact and the expression of vimentin was relatively low, similar to levels observed in control corneas (Figure 2C). At 7 days after injury, levels of vimentin-immunoreactivity remained elevated, similar to levels observed at 4 days after injury (Figure 2C,D). Levels of vimentin-immunoreactivity remained elevated in stromal cells at 14 days after treatment, but the stromal cells that expressed high levels of vimentin appeared to spread laterally away from the site of incision (Figure 2E). Vimentin was not observed in the corneal epithelium at any time point after corneal incision.

We next examined the expression of vinculin, a membrane-associated cytoskeletal protein, in damaged corneas. In control corneas, vinculin was expressed at low levels in stromal cells, whereas vinculin was not detected in the epithelium (Figure 2F) or in the endothelium (not shown). One day after injury, vinculin expression in the stromal cells remained unchanged (Figure 2G). There was no vinculin-immunofluorescence observed in the regenerated epithelium overlying the corneal wound at any time point after injury (Figure 2G-J). At 4 days after injury, there was a dramatic increase in the expression of vinculin in stromal cells within the region of corneal incision (Figure 2H). Vinculin expression remained elevated in the wound-activated stromal cells through 7 days after injury (Figure 2I). By 14 days after injury, the expression of vinculin was decreased compared to expression levels seen at 7 days after injury (Figure 2I,J). However, levels of vinculin expression remained elevated at 14 days after injury compared to levels seen in controls, and appeared to spread laterally away from the site of incision compared to the distribution of reactive cells seen at earlier time points (Figure 2J).

**Figure 3:**
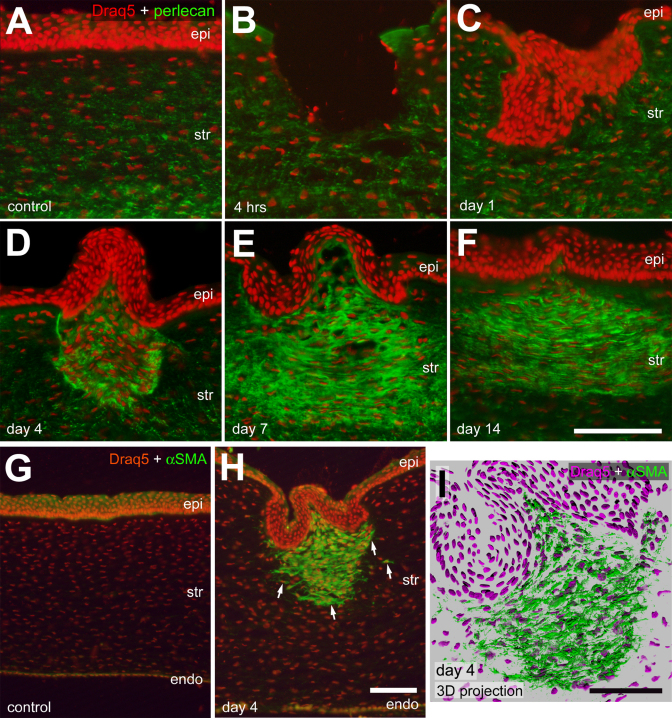
α-Smooth muscle actin (αSMA) and perlecan are upregulated in the stroma following corneal injury. Transverse sections of the control (**A** and** G**) and treated (**B**-**F**, **H** and **I**) corneas were labeled with Draq5 (red nuclei) and antibodies perlecan (**A**-**F**; green) or αSMA (**G**-**I**; green). Corneas were harvested from control eyes (**A** and **G**) or 4 h (**B**), 1 day (**C**), 4 days (**D**, **H**, and **I**), 7 days (**E**), and 14 days (**F**) after injury. **I** is a 3D shadow reconstruction of a z-series of confocal optical sections to demonstrate the horizontal arrangement of presumptive myofibroblasts in the stroma within injured region. Arrows indicate cells labeled for αSMA and Draq5. The calibration bar (50 µm) in **F** applies to **A**-**F**, the bar in **H** applies to **G** and **H**, and the bar in **I** applies to **I** alone. Abbreviations: epi – epithelium, str – stroma, endo – endothelium.

We next probed for the expression of perlecan, a heparan sulfate proteoglycan that is associated with the endothelial basement membrane in the cornea [19,20]. In untreated corneas, perlecan was observed in the posterior cornea near Descemet’s membrane (Figure 3A) and at high levels in the endothelium (data not shown), consistent with previous reports [21,22]. Perlecan-immunofluorescence was detected at relatively low levels in the anterior stroma, and was not detected in Bowman’s membrane or the epithelium (Figure 3A). At 4 h and 1 day after injury, perlecan expression in the stroma was unchanged compared to that seen in control corneas (Figure 3A-C). At 4 days injury, perlecan expression was upregulated in stromal cells within the site of incision, corresponding to the area of increased cell density (Figure 3D). At 7 days after injury, perlecan remained upregulated in stromal cells in the area of the wound (Figure 3E). This focal upregulation of perlecan was observed at 14 days after, but appeared to spread laterally away from the site of incision (Figure 3F). We failed to detect perlecan in the corneal epithelium, which is not consistent with reports of perlecan in the cornea of chicks and other species. The 5C9 monoclonal antibody to perlecan recognizes domain IV. It is possible that a splice variant of perlecan that excludes domain IV is present in the epithelium of the chick cornea, and, therefore, was not detected in our studies.

Antibodies to alpha-smooth muscle actin (αSMA) were used to examine injury-induced changes in keratocytes after corneal injury. In control chick corneas, αSMA was not observed (Figure 3G). At 1 day after injury, αSMA was not detectable in stromal cells (not shown). By contrast, 4 days after injury αSMA was markedly upregulated in the stromal cells immediately under the area of incision (Figure 3H,I). By using confocal microscopy and 3D reconstruction, we found αSMA surrounding the nuclei of activated corneal fibroblast-like cells (Figure 3I). αSMA-expressing stromal cells were present immediately posterior to Bowman’s membrane and extended approximately 200 µm into the stroma (Figure 3H-I). Increased levels of α-SMA-immunofluoresence continued through 7 and 14 days after injury (not shown).

### Invasion of immune cells into injured corneas

**Figure 4:**
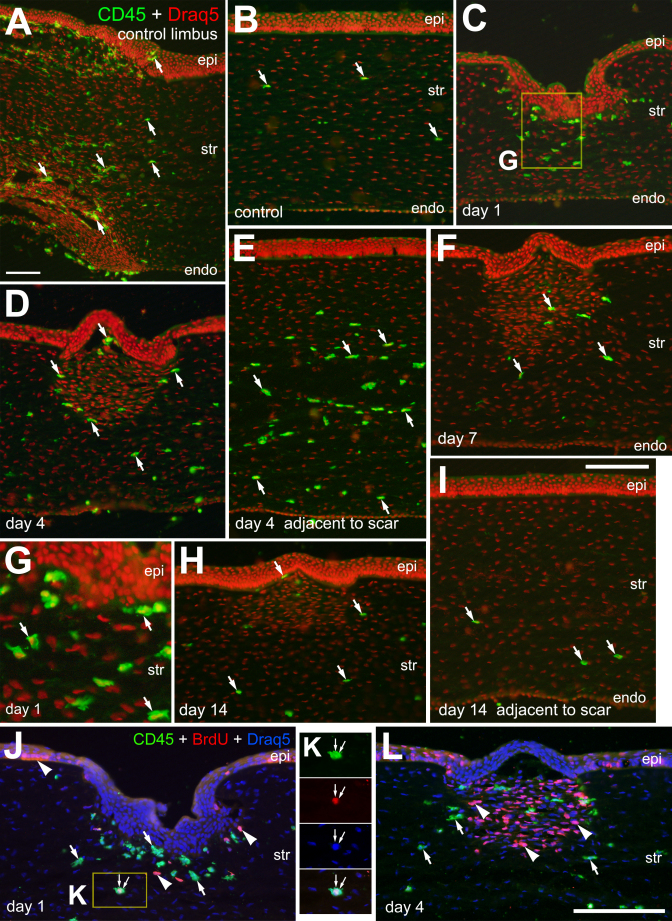
CD45+ monocytes accumulate in the stroma following corneal injury. Transverse section of undamaged (**A** and **B**) and injured (**C**-**L**) corneas were labeled with Draq5 (red nuclei in **A**-**I**; blue nuclei **J** and **L**) and antibodies to CD45 (green) and BrdU (red in **J** and **L**). Corneas were harvest at 1 (**C**, **G**, and **J**), 4 (**D**, **E**, and **L**), 7 (**F**), and 14 (**H** and **I**) days after injury. Panel **G** is a 2.5 fold enlargement of the area (yellow box) in **C** indicating the amoeboid morphology of CD45+ monocytes in the site of injury in the stroma. Panel **K** is a 1.5 fold enlargement of the area (yellow box) in **J** providing a single-channel images and a merged overlay of labeling for CD45, BrdU and Draq5 in a single monocyte. Arrows indicate cells labeled for CD45 and Draq5, arrowheads indicate BrdU-labeled cells (**J** and **L**), and small double-arrows indicate a single cell labeled for CD45, BrdU and Draq5 (**J** and **K**). The calibration bar (50 µm) in panel **A** applies to **A** alone, the bar in **I** applies to **B**-**I**, and the bar in **L** applies to **J** and **L**. Abbreviations: epi – epithelium, str – stroma, endo – endothelium.

Activated macrophages are known to accumulate and participate in the wound healing response of the stroma in damaged corneas [23] (reviewed by [4]). To identify monocytes that invade the cornea we applied antibodies to CD45. In undamaged corneas, we identified numerous CD45+ cells scattered across the limbal region of the cornea (Figure 4A). There were few CD45+ cells in the stroma of undamaged corneas (Figure 4B). At 1 day after injury, there was a significant accumulation of CD45+ cells in anterior regions of the stroma, surrounding the site of incision (Figure 4C). The CD45+ cells appeared to be reactive macrophages with high levels of CD45-expression and ameboid morphophology (Figure 4C,G) At 4 days after injury, there were many CD45+ cells remaining within the stroma at the site of injury (Figure 4D) and scattered throughout the stroma in regions adjacent to the site of incision, across the entire cornea (Figure 4E). Within the site of injury at 7 and 14 days after incision, the abundance of CD45+ cells appeared diminished compared to numbers seen at day 4 (Figure 4F,H). At 14 days after injury, the abundance of CD45+ cells in the stroma outside of the injured region was reduced to levels seen in undamaged corneas (Figure 4B,I). To determine whether any of the CD45+ monocytes that accumulate in the stroma are derived through proliferation, we probed for BrdU-labeling at 1 (BrdU injected 4 h before harvest) and 4 days (BrdU injected at days 1, 2, and 3). We found very few of the CD45+ positive monocytes that accumulate within the stroma in response to injury were labeled for BrdU (Figure 4J,K). At 4 days after incision, by comparison, we found that none of the BrdU-labeled cells that accumulate within the injury zone of the stroma were CD45+ monocytes (Figure 4L).

### Corneal innervation after corneal incision

**Figure 5:**
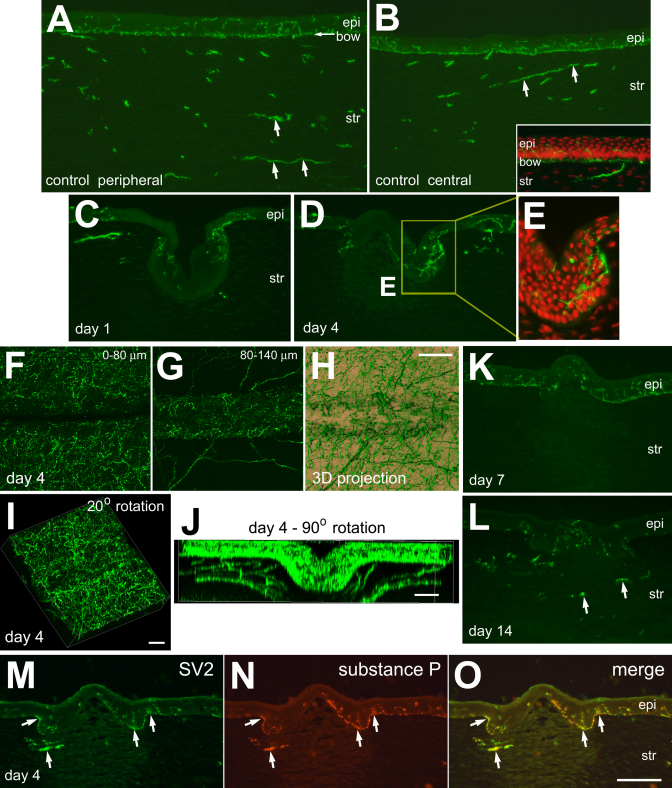
Neuronal re-innervation of the corneal epithelium occurs rapidly after injury. Transverse sections (**A**-**E**, **H**-**O**) or whole-mount preparations (**F**-**J**) were labeled with Draq5 (red) or antibodies to SV2 (green) and substance P (red; **N** and **O**). The inset in **B** is a high magnification view of SV2-positive neuronal ending in the basal layers of the corneal epithelium. Panel **E** is a twofold enlargement of the boxed-out (yellow rectangle) area in **D**. Panel **F** is a projection of confocal Z-stack of images taken from the surface of the epithelium down to 80 µm of depth. Panel **G** is a projection of confocal Z-stack of images from 80 µm down to 140 µm of depth from the surface of the cornea. Panel **H** is a 3D shadow reconstruction to demonstrate the arrangement of SV2-positive nerve terminals re-innervating regenerated epithelium. Panels **I** and **J** are 20° and 90° rotations of confocal Z-stack reconstructions to demonstrate the re-innervation of the trough of regenerated epithelium within the corneal incision. Arrows indicate SV2/substance P-positive axons in the stroma. The calibration bar (50 µm) in panel **O** applies to panels **A**-**D**, **K**, **L**, and **M**-**O.** The bar in **H** applies to all **F**-**H** panels, the bar in **I** applies to **I** alone, the bar in **J** applies to **J** alone. Abbreviations: epi – epithelium, str – stroma, endo – endothelium.

Branches and terminals of the ophthalmic branch of the trigeminal nerve (Cranial Nerve V_1_) in the cornea were labeled using antibodies to SV2, a synaptic vesicle protein. Examination of control corneas revealed extensive neuronal processes running in the basal layers of the epithelium in peripheral and central regions of the cornea (Figure 5A,B). The SV2-labeled neuronal processes appeared to run in bundles of axons in deeper layers of the stroma near the limbus (Figure 5A). The axons in the stroma appeared to ascend and terminate with numerous dense endings in basal layers of the epithelium (Figure 5A,B). At 1 day after corneal incision, few neuronal processes were observed in the basement membrane and epithelium overlying the wound (Figure 5C). There were neuronal processes at the edge of the site of incision in the regenerated epithelium, whereas there were no processes in the stroma immediately underlying the injury (Figure 5C). At 4 days after injury, numerous SV2-positive endings were present in the regenerated epithelium at the edge of the incision and within the trough of the incision (Figure 5D-J). At 7 days after injury, SV2-positive processes were observed in the epithelium overlying the site of injury (Figure 5K). At 14 days after injury, SV2-positive neuronal processes, that were presumably regenerated, were observed within the injured stromal region where the cell density was elevated (Figure 5L).

The neuropeptide substance P has been reported to be released from the axon terminals that innervate the cornea, and this peptide is known to regulate the proliferation and turn-over of corneal epithelial cells (reviewed by [24]). Thus, we probed for substance P in axons and nerve terminals that re-innervate damaged regions of cornea. We found substance P-immunoreactivity in all SV2-positive axons in the stroma and terminals in the epithelium in undamaged corneas (data not shown). In addition, we found substance P-immunoreactivity in the SV2-positive terminals in the regenerated epithelium that filled the injury site at 4 days after incision (Figure 5M-O).

### Cell death after corneal injury

**Figure 6:**
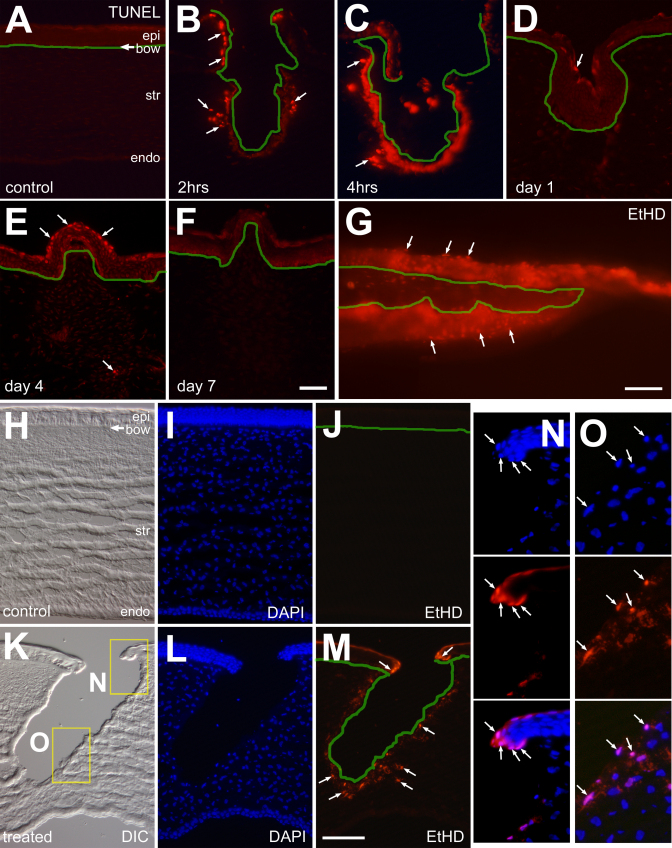
Corneal incision induces the death of epithelial and stromal cells shortly after injury. Transverse sections of the cornea were labeled for TUNEL (**A**-**F**), DAPI (**I**, **L**, **N**, and **O**), or EtHD (**J**, **M**, **N**, and **O**). Corneas were obtained at 15 min (**G** and **K**-**O**), 2 h (**B**), 4 h (**C**), 1 day (**D**), 4 days (**E**) or 7 days (**F**) after injury. The green lines indicate Bowman’s membrane and the edge of the incision. Panel **G** is a whole-mount preparation of an acutely injured cornea that was labeled with EtHD. Panels **H** and **K** are DIC images of control and damaged corneas. Panels **N** and **O** are 2.5 fold enlargements of the boxed-out (yellow rectangles) areas in panel **K**. Arrows indicate the nuclei of dying cells. The calibration bar (50 µm) in panel **F** applies to panels **A**-**F**, the bar in panel in **G** applies to **G** alone and the bar in panel **M** applies to panels **H**-**M**. Abbreviations: epi – epithelium, str – stroma, endo – endothelium.

Examination of undamaged corneas revealed no TUNEL-positive cells in the stroma, endothelium or epithelium (Figure 6A). After injury, TUNEL-positive cells were observed in the corneal epithelium and within the stroma. At 2 h after injury, numerous TUNEL-positive cells were observed lining the border of the corneal incision, within the epithelium and stroma that flanked the site of incision (Figure 6B). Numbers of TUNEL-labeled cells appeared increased at 4 h after injury (Figure 6C). At 1 day after injury, few TUNEL-positive cells were observed in the regenerated epithelium that filled-in the site of incision, and no TUNEL-positive cells were observed within the underlying stroma (Figure 6D). Four days after injury, we observed TUNEL-positive cells in the corneal epithelium overlying the wound (Figure 6E). Most of the TUNEL-positive cells were found in the superficial layers of the epithelium overlying the site of injury (Figure 6E). Very few TUNEL-positive cells were observed in the deep layers of the stroma within the region of the incision (Figure 6E). At 7 days after injury, TUNEL-positive cells were not observed in the epithelium or the stroma in damaged corneas (Figure 6F).

To compliment the data from the TUNEL studies, we applied ethidium homodimer (EtHD) shortly (~15 min) after injury. In undamaged corneas, EtHD does not label any cells within the epithelium, stroma or endothelium (Figure 6H-J). By contrast, we identified numerous dead or dying epithelial cells, labeled with EtHD, immediately surrounding the site of injury (Figure 6G,K-N). In addition, we found a few EtHD-labeled cells within the stroma at the edge of the incision site and extending as far as 40 µm into the stroma (Figure 6K-M,O). Labeling with EtHD suggests that epithelial cells and stromal cells die of necrosis shortly after injury. We failed to find apoptotic cells immunolabeled for cleaved caspase 3 (CC3) within injured corneas at any time after damage (data not shown).

## Discussion

Consistent with reports in other species, we find that corneal cells undergo dramatic changes during recovery from an incision. These changes involve the epithelial and stromal cells that surround the site of injury, neuronal re-innervation and monocytes across the injured cornea. The most pronounced cellular changes in response to injury were observed within the stroma. The corneal stroma comprises organized lamellar sheets of type I and V collagen, proteoglycans, glycoseaminoglycans (GAGs), and interspersed quiescent keratocytes. In normal corneas, keratocytes have a flattened morphology with numerous gap junctions, forming a syncytium of cells between the lamellar sheets (reviewed by [3]). Keratocytes act as the caretakers of the corneal stroma, with turnover of these cells and the extracellular matrix occurring at very low rate compared to other tissues. After injury, the cornea shows a well regulated, coordinated response to re-establish tissue integrity and function. Upon damage to the epithelium, keratocytes are exposed to several wound healing modulators produced by corneal epithelial cells, such as Interleukin-1 (IL-1), Interleukin-6 (IL-6) and transforming growth factor-beta (TGF-β). Exposure to these factors can induce apoptosis in keratocytes in the region immediately surrounding the corneal wound, and stimulate the proliferation and migration of nearby cells to fill-in the site of incision (reviewed by [4]). These keratocytes transdifferentiate into fibroblasts, migrate into the wound, and secrete matrix metalloproteinases, collagenases and gelatinases to degrade the damaged collagen and extracellular matrix (ECM) components. These fibroblasts secrete new ECM to fill and remodel the cornea. Keratocytes may also differentiate into myofibroblasts, which express α-SMA, have contractile properties, and secrete ECM components to promote wound healing [5,25] (reviewed by [3,4,26]). αSMA is known to upregulated in corneal keratocytes in a rodent model of corneal injury [27]. We found that wound healing involved deposition of the ECM proteoglycan perlecan in the stroma within the region of injury. Consistent with our findings, accumulations of perlecan in the stroma have been observed following corneal injury [16,28-30]. It is worth noting that the cellular responses that we observed were made on animals that were between 1 and 4 weeks of age, and the responses of corneal cells in older chicks may be different.

In response to injury, we find significant changes in the expression of cytoskeletal filaments by reactive keratocytes within the zone of injury. The cytoskeleton is composed of 3 classes of cellular filaments: 1) actin-based microfilaments, 2) microtubules and 3) intermediate filaments. Intermediate filaments serve as a cellular scaffold that protects cells from mechanical stress (reviewed by [31,32]). Intermediate filaments are divided into 5 subclasses, with vimentin classified as a type-III intermediate filament expressed in mesenchymal and some ectodermal derived cells during development (reviewed by [33]). In the stroma, vimentin is expressed at low levels in undamaged corneas. After injury, the levels of vimentin expressed in the stroma at the site of injury increase by day 4 and remain increased through day 14. The elevated levels of vimentin expression correspond to the hypercellularity of the stroma, consistent with findings in different models of corneal injury [34-36]. The transient increases in filamentous proteins expressed by stromal keratocytes within the region of injury likely function to enhance the structural properties of cells to physically bridge a wound and/or enhance the migration of cells into the wound.

Our findings indicate a rapid regeneration of the epithelium shortly after corneal incision. We did not find BrdU-labeled cells within the regenerated epithelium when the BrdU was applied at 20 h after injury, and we did not find any indication of epithelial regeneration at 4 h after incision. However, we cannot exclude the possibility that a “burst” of proliferation occurs between 4 and 20 h after incision to contribute to the epithelial cells that fill the surface of the wound. We propose that the epithelial cells that fill the wound migrate into the trough from regions flanking the site of injury, consistent with reports from other animal models that the re-establishment of epithelial layers following injury results primarily from movement of nearby cells into the wound [37-40]. Only when BrdU was injected at 7 and 8 days after injury did we observe significant numbers of proliferating cells in basal layers of the epithelium that covered the wound, suggesting that re-establishment of local proliferation and turn-over epithelial cells occurs at least 1 week after injury.

Among the types of cells that migrate into the wound are pro-inflammatory immune cells such as polymorphonuclear leukocytes and activated macrophages (reviewed by [4]). We find that there is a rapid, transient accumulation of CD45-positive monocytes in damaged corneas. These CD45-positive cells were most likely to be activated macrophages given that these cells had an amoeboid morphology and elevated levels of CD45 [41,42]. The accumulation of the putative macrophages were prominent within the site of injury, but also included increased numbers of macrophages across the entire cornea following a focal incision. We detected few BrdU-labeling CD45-positive cells in damaged corneas at 1 day after treatment, suggesting that few of the monocytes proliferate within the stroma in response to injury. Collectively, these findings suggest that a focal incision attracts monocytes from limbal regions of the cornea, which normally contains numerous CD45-positive monocytes, to migrate into the site of stromal incision.

Similar to the innervation of the cornea other species, the avian cornea is innervated by the ophthalmic branch of the trigeminal nerve [43]. Proper corneal innervation provides a high-density network of touch sensitive nerve endings that are required to maintain corneal clarity and integrity; loss of innervation causes a disintegration of the epithelium [44-48]. We find that the neuronal re-innervation of the injured cornea was robust, occurred rapidly (<4 days), unlike re-innervation of the stroma which occurs more slowly (>7 days). In addition, we find that substance P is present in the nerve terminals that re-innervate the epithelial cells that fill the wound. This is consistent with findings that substance P plays important roles regulating the integrity and turn-over of corneal epithelial cells [49-52]. The corneal epithelium regenerated in less than 24 h to fill-in the site of the incision. Our data indicate that neuronal re-innervation followed shortly after regeneration of the epithelium. The rapid re-innervation likely occurred through sprouting and re-growth of axons and terminals. We propose that the chick cornea is an excellent models system in which to study axon sprouting and regeneration following injury.

We find that corneal wound healing involves a long-lasting increase in the density of stromal cells within the region of the incision. Our findings suggest that the increase in cell density occurs, at least in part, from proliferation of local keratocytes within the stroma. Consistent with this hypothesis, we failed to detect a significant decrease in cell density in stromal regions flanking the wound, suggesting there was not a significant migration of keratocytes from adjacent stromal regions into the wound. However, we cannot exclude the possibility that stromal cells migrated into wound from areas lateral to the incision. Other studies have demonstrated an accumulation of stromal cells within wounds and wound-induced proliferation of keratocytes [27,53-55].

Our data suggest that delayed, apoptotic cell death accounts for relatively little cell loss within the cornea following an incision. We find numerous dying cells in the epithelium and epithelium immediately flanking the site of incision between 15 min and 4 h of injury. These findings are consistent with necrotic cell death underlying the loss of the cells shortly after insult, which is known to occur shortly after injury and can be detected by labeling with TUNEL or EtHD. We failed to detect apoptotic corneal cells that were immunoreactive for CC3. By comparison, this antibody to CC3 effectively labels apoptotic ganglion cells in damaged chick retina [56], supporting the specificity of this antiserum in chick tissues. Other models of corneal wound healing have demonstrated significant numbers of apoptotic cells within the stroma many days after injury [57,58](reviewed by [59]).

### 

#### Conclusions

We find that the chick cornea is amenable to experimental manipulations. Reproducible incisions were made to the cornea and stereotypical wound healing responses were observed within the stroma and epithelium. Following rapid epithelial regeneration and neuronal re-innervation of the epithelium, we find an accumulation of proliferating stromal cells within the corneal wound, and a transient accumulation of monocytes across injured corneas with concentrations at the site of incision. The reactive stromal cells expressed elevated levels of filamentous cytoskeletal proteins and extracellular matrix molecules. We propose that the chick cornea may serve as an outstanding model system in which to study axonal regeneration and cell-signaling pathways that regulate wound healing.
